# Veratrine in Rheumatism

**Published:** 1854-02

**Authors:** 


Veratrine in Rheumatism.—Some marvellous results have followed the use of veratrine in articular diseases. The trial was made by M. Trousseau, who gave the tenth of a grain in a pill the first day; two on the second; three on the third, and so on increasing the dose, one pill each day, until it reached six or seven. Its action appears to be most decided in breaking down the inflammatory action. It is maintained that it has anti-rheumatic virtues and from its influence over the pulse it may be found by further trial, to be a most valuable therapeutic agent in all these arthrodial affections.—*Iowa Medical Journal.*

